# Methyl (*Z*)-2-[(2,4-dioxothia­zolidin-3-yl)meth­yl]-3-(2-methyl­phen­yl)prop-2-enoate

**DOI:** 10.1107/S1600536811053682

**Published:** 2011-12-17

**Authors:** S. Vijayakumar, S. Murugavel, D. Kannan, M. Bakthadoss

**Affiliations:** a Department of Physics, Sri Balaji Chokkalingam Engineering College, Arni, Thiruvannamalai 632 317, India; b Department of Physics, Thanthai Periyar Government Institute of Technology, Vellore 632 002, India; c Department of Organic Chemistry, University of Madras, Maraimalai Campus, Chennai 600 025, India

## Abstract

The C=C bond in the title compound, C_15_H_15_NO_4_S, has a *Z* configuration. The thia­zolidine ring is essentially planar [maximum deviation = 0.008 (1) Å for the N atom] and is oriented at a dihedral angle of 59.1 (1)° with respect to the benzene ring. In the crystal, pairs of C—H⋯O hydrogen bonds link centrosymmetrically related mol­ecules into dimers, generating *R*_2_^2^(18) ring motifs. The crystal packing is further stabilized by C—H⋯π and C—O⋯π [O⋯centroid = 3.412 (2) Å and C—O⋯centroid = 115.0 (1)°] inter­actions.

## Related literature

For the biolgical activity of thia­zolidine derivatives, see: Chen *et al.* (2000 ▶); Jacop & Kutty (2004 ▶); Kalia *et al.* (2007 ▶); Vicentini *et al.* (1998 ▶); Vigorita *et al.* (1992 ▶). For resonance effects of acrylate, see: Merlino (1971 ▶); Varghese *et al.* (1986 ▶). For closely related structures, see: Fun *et al.* (2009 ▶); Vennila *et al.* (2011 ▶). For hydrogen-bond motifs, see: Bernstein *et al.* (1995 ▶).
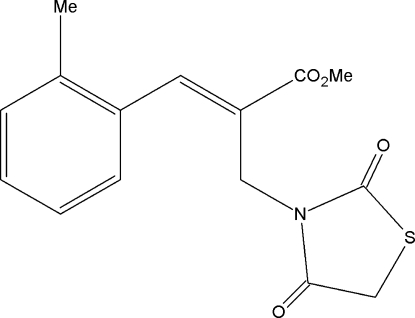


## Experimental

### 

#### Crystal data

C_15_H_15_NO_4_S
*M* *_r_* = 305.34
Monoclinic, 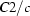

*a* = 21.3744 (10) Å
*b* = 6.9762 (3) Å
*c* = 20.3084 (10) Å
β = 103.361 (2)°
*V* = 2946.3 (2) Å^3^
*Z* = 8
Mo *K*α radiation
μ = 0.23 mm^−1^
*T* = 293 K
0.26 × 0.23 × 0.18 mm

#### Data collection

Bruker APEXII CCD diffractometer
Absorption correction: multi-scan (*SADABS*; Sheldrick, 1996 ▶) *T*_min_ = 0.941, *T*_max_ = 0.959
18757 measured reflections
4354 independent reflections
2966 reflections with *I* > 2σ(*I*)
*R*_int_ = 0.027

#### Refinement

*R*[*F* ^2^ > 2σ(*F* ^2^)] = 0.045
*wR*(*F* ^2^) = 0.135
*S* = 1.04
4354 reflections
192 parameters
H-atom parameters constrained
Δρ_max_ = 0.26 e Å^−3^
Δρ_min_ = −0.32 e Å^−3^

### 

Data collection: *APEX2* (Bruker, 2004 ▶); cell refinement: *APEX2* and *SAINT* (Bruker, 2004 ▶); data reduction: *SAINT* and *XPREP* (Bruker, 2004 ▶); program(s) used to solve structure: *SHELXS97* (Sheldrick, 2008 ▶); program(s) used to refine structure: *SHELXL97* (Sheldrick, 2008 ▶); molecular graphics: *ORTEP-3* (Farrugia, 1997 ▶); software used to prepare material for publication: *SHELXL97* and *PLATON* (Spek, 2009 ▶).

## Supplementary Material

Crystal structure: contains datablock(s) global, I. DOI: 10.1107/S1600536811053682/bt5747sup1.cif

Structure factors: contains datablock(s) I. DOI: 10.1107/S1600536811053682/bt5747Isup2.hkl

Supplementary material file. DOI: 10.1107/S1600536811053682/bt5747Isup3.cml

Additional supplementary materials:  crystallographic information; 3D view; checkCIF report

## Figures and Tables

**Table 1:** Hydrogen-bond geometry (Å, °) *Cg*1 is the centroid of the C7–C12 benzene ring.

*D*—H⋯*A*	*D*—H	H⋯*A*	*D*⋯*A*	*D*—H⋯*A*
C8—H8⋯O2^i^	0.93	2.55	3.379 (2)	148
C9—H9⋯*Cg*1^ii^	0.93	2.90	3.677 (2)	142

Symmetry codes: (i) 
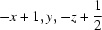
; (ii) 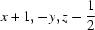.

**Fig. 1.**
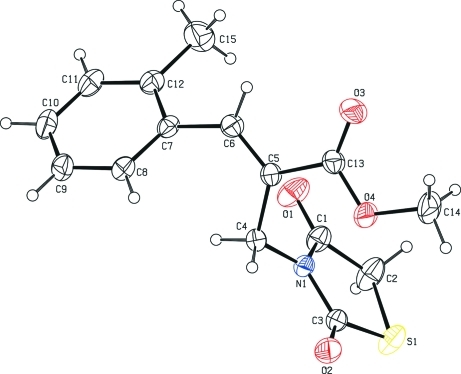
The molecular structure of the title compound with the atom numbering scheme. Displacement ellipsoids are drawn at the 30% probability level. H atoms are presented as a small spheres of arbitrary radius.

**Fig. 2.**
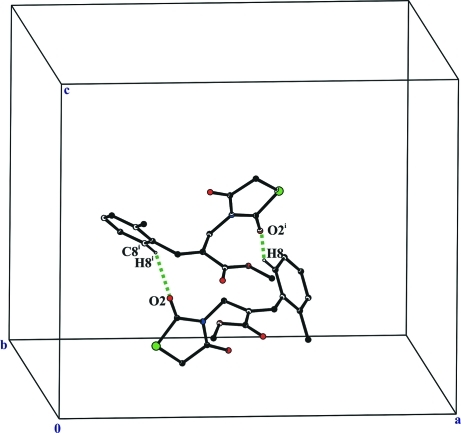
Part of the crystal structure of the title compound showing C—H···O intermolecular hydrogen bonds (dotted lines) generating R22(18) centrosymmetric dimer. [Symmetry code: (i) 1-x, y, 1/2-z].

**Fig. 3.**
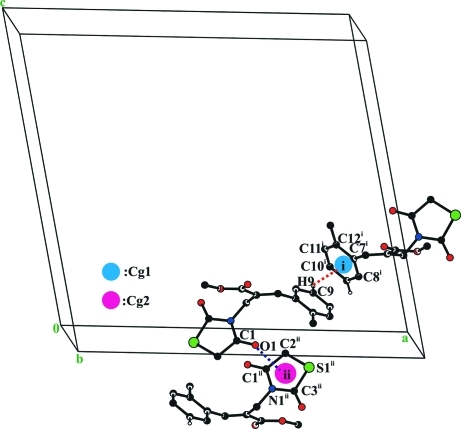
A view of the C—H···π (red dotted lines) and C—O···π (blue dotted lines) interactions, in the molecular structure of the title compound. Cg1 and Cg2 are the centroids of the (C7–C12) benzene ring and (N1/S1/C1–C3) thiazolidine ring, respectively. [Symmetry code: (ii) 3/2-x, 1/2+y, 1/2-z; (iii) 1-x, 2-y, -z.]

## supplementary crystallographic information

### Comment

Thiazolidine derivatives exhibit herbicidal (Chen *et al.,* 2000; Vicentini *et al.,* 1998),  antineoplastic (Vigorita *et al.,* 1992), hypolipidemic (Jacop & Kutty, 2004) and anti-inflammatory (Kalia *et al.,* 2007) activities. In view of this importance, the crystal structure of the title compound has been carried out and the results are presented here.

Fig. 1. shows a displacement ellipsoid plot of (I), with the atom numbering scheme. The molecules of the title compound display a *Z*– configuration about the C6═C5 double bond. The thiazolidine moiety (S1/N1/C1-C3) is essentially planar [maximum deviation = -0.008 (1) Å for the N1 atom] and lies at an angle 59.1 (1)° with respect to the benzene ring. The significant difference in length of the C13—O4 = 1.334 (2) Å and C14—O4 = 1.446 (2) Å bonds is attributed to a partial contribution from the O^-^–C = O^+^–C resonance structure of the O3═C13—O4—C14 group (Merlino, 1971). This feature, commonly observed in the carboxylic ester group of the substituents in various compounds gives average values of 1.340 Å and 1.447 Å respectively for these bonds (Varghese *et al.,* 1986). The sum of bond angles around N1 (360°) indicates that N1 is in *sp*^2^ hybridization. The geometric parameters of the title molecule agrees well with those reported for similar structures (Fun *et al.,* 2009, Vennila *et al.,* 2011).

In the crystal packing (Fig. 2), the molecules at *x, y, z* and *1-x, y, 1/2-z* are linked by C8—H8···O2 hydrogen bonds into cyclic centrosymmetric *R_2_^2^*(18) dimers (Bernstein *et al.,* 1995). The crystal packing is further stabilized by C—H···π and C—O···π interactions, the first one between a H9 atom and neighbouring benene ring (C7–C12), with a C9—H9···Cg1^ii^ seperation of 2.90 Å (Fig. 3 and Table 1; Cg1 is the centroid of the C7–C12 benzene ring, Symmetry code as in Fig.3), and the second one between oxygen atom O1 and neighbouring thiazolidine ring (N1/S1/C1–C3), with a Ol···centroid(Cg2^iii^) distance of 3.412 (2) Å and a C1—O1···Cg2^iii^ angle of 115.0 (1)° (Fig. 3; Cg2 is the centroid of the N1/S1/C1—C3 thiazolidine ring, Symmetry code as in Fig. 3).

### Experimental

A solution of thiazolidine-2,4-dione (1 mmol, 0.117 g) and potassium carbonate (1.5 mmol, 0.207 g) in acetonitrile solvent was stirred for 15 minutes at room temperature. To this solution, (*Z*)-methyl-2 -(bromomethyl)-3-(2-methylphenyl)-prop-2-enoate (1 mmol, 0.269 g) was added dropwise till the addition is complete. After the completion of the reaction, as indicated by TLC, acetonitrile was evaporated. Ethyl acetate (15 ml) and water (15 ml) were added to the crude mass. The organic layer was dried over anhydrous sodium sulfate. Removal of solvent led to the crude product, which was purified through pad of silica gel (100–200 mesh) using ethylacetate and hexanes (1:9) as solvents. The pure title compound was obtained as a colourless solid (0.290 g, 95% yield). Recrystallization was carried out using ethylacetate as solvent.

### Refinement

H atoms were positioned geometrically, with C—H = 0.93–0.97 Å and constrained to ride on their parent atom, with *U*_iso_(H)=1.5*U*_eq_ for methyl H atoms and 1.2*U*_eq_(C) for other H atoms.

### Crystal data

**Table:**

C_15_H_15_NO_4_S	*F*(000) = 1280
*M**_r_* = 305.34	*D*_x_ = 1.377 Mg m^−^^3^
Monoclinic, *C*2/*c*	Mo *K*α radiation, λ = 0.71073 Å
Hall symbol: -C 2yc	Cell parameters from 4370 reflections
*a* = 21.3744 (10) Å	θ = 2.0–30.2°
*b* = 6.9762 (3) Å	µ = 0.23 mm^−^^1^
*c* = 20.3084 (10) Å	*T* = 293 K
β = 103.361 (2)°	Block, colourless
*V* = 2946.3 (2) Å^3^	0.26 × 0.23 × 0.18 mm
*Z* = 8	

### Data collection

**Table:**

Bruker APEXII CCD diffractometer	4354 independent reflections
Radiation source: fine-focus sealed tube	2966 reflections with *I* > 2σ(*I*)
graphite	*R*_int_ = 0.027
Detector resolution: 10.0 pixels mm^-1^	θ_max_ = 30.2°, θ_min_ = 2.0°
ω scans	*h* = −29→30
Absorption correction: multi-scan (*SADABS*; Sheldrick, 1996)	*k* = −9→8
*T*_min_ = 0.941, *T*_max_ = 0.959	*l* = −28→18
18757 measured reflections	

### Refinement

**Table:**

Refinement on *F*^2^	Primary atom site location: structure-invariant direct methods
Least-squares matrix: full	Secondary atom site location: difference Fourier map
*R*[*F*^2^ > 2σ(*F*^2^)] = 0.045	Hydrogen site location: inferred from neighbouring sites
*wR*(*F*^2^) = 0.135	H-atom parameters constrained
*S* = 1.04	*w* = 1/[σ^2^(*F*_o_^2^) + (0.0612*P*)^2^ + 1.2153*P*] where *P* = (*F*_o_^2^ + 2*F*_c_^2^)/3
4354 reflections	(Δ/σ)_max_ < 0.001
192 parameters	Δρ_max_ = 0.26 e Å^−^^3^
0 restraints	Δρ_min_ = −0.32 e Å^−^^3^

### Special details

**Table:**

Geometry. All esds (except the esd in the dihedral angle between two l.s. planes) are estimated using the full covariance matrix. The cell esds are taken into account individually in the estimation of esds in distances, angles and torsion angles; correlations between esds in cell parameters are only used when they are defined by crystal symmetry. An approximate (isotropic) treatment of cell esds is used for estimating esds involving l.s. planes.
Refinement. Refinement of F^2^ against ALL reflections. The weighted R-factor wR and goodness of fit S are based on F^2^, conventional R-factors R are based on F, with F set to zero for negative F^2^. The threshold expression of F^2^ > 2sigma(F^2^) is used only for calculating R-factors(gt) etc. and is not relevant to the choice of reflections for refinement. R-factors based on F^2^ are statistically about twice as large as those based on F, and R- factors based on ALL data will be even larger.

### Fractional atomic coordinates and isotropic or equivalent isotropic displacement parameters (Å^2^)

**Table:**

	*x*	*y*	*z*	*U*_iso_*/*U*_eq_	
S1	0.34666 (2)	0.81404 (9)	0.02048 (3)	0.06438 (17)	
N1	0.46454 (5)	0.81887 (16)	0.09106 (6)	0.0362 (3)	
C4	0.51881 (7)	0.82904 (19)	0.14998 (8)	0.0383 (3)	
H4A	0.5026	0.8529	0.1901	0.046*	
H4B	0.5460	0.9364	0.1444	0.046*	
C3	0.40174 (7)	0.8361 (2)	0.09848 (9)	0.0414 (3)	
O4	0.46444 (5)	0.48419 (16)	0.16471 (7)	0.0588 (3)	
C6	0.62202 (7)	0.6461 (2)	0.16616 (8)	0.0414 (3)	
H6	0.6416	0.5262	0.1714	0.050*	
C7	0.66451 (6)	0.8118 (2)	0.16498 (7)	0.0387 (3)	
O2	0.38694 (6)	0.86509 (19)	0.15080 (7)	0.0583 (3)	
C8	0.66505 (7)	0.9661 (2)	0.20808 (8)	0.0435 (3)	
H8	0.6368	0.9677	0.2367	0.052*	
O3	0.55140 (6)	0.30807 (16)	0.16562 (8)	0.0638 (4)	
C13	0.52619 (7)	0.4616 (2)	0.16372 (8)	0.0432 (3)	
C5	0.55872 (7)	0.64927 (19)	0.16051 (7)	0.0378 (3)	
C1	0.47255 (8)	0.7930 (2)	0.02682 (8)	0.0473 (4)	
O1	0.52437 (6)	0.7789 (2)	0.01315 (7)	0.0677 (4)	
C12	0.70731 (7)	0.8089 (2)	0.12198 (9)	0.0463 (4)	
C11	0.74813 (8)	0.9646 (3)	0.12352 (9)	0.0542 (4)	
H11	0.7760	0.9662	0.0945	0.065*	
C9	0.70709 (8)	1.1182 (3)	0.20911 (9)	0.0522 (4)	
H9	0.7072	1.2207	0.2384	0.063*	
C10	0.74848 (8)	1.1161 (3)	0.16660 (10)	0.0563 (4)	
H10	0.7768	1.2176	0.1670	0.068*	
C2	0.40897 (9)	0.7833 (4)	−0.02463 (9)	0.0668 (6)	
H2A	0.4044	0.6605	−0.0476	0.080*	
H2B	0.4066	0.8838	−0.0581	0.080*	
C14	0.42702 (10)	0.3104 (3)	0.16151 (13)	0.0740 (6)	
H14A	0.4213	0.2544	0.1173	0.111*	
H14B	0.3858	0.3400	0.1701	0.111*	
H14C	0.4491	0.2214	0.1949	0.111*	
C15	0.70806 (12)	0.6449 (3)	0.07429 (13)	0.0782 (7)	
H15A	0.6691	0.6460	0.0394	0.117*	
H15B	0.7112	0.5262	0.0987	0.117*	
H15C	0.7443	0.6578	0.0542	0.117*	

### Atomic displacement parameters (Å^2^)

**Table:**

	*U*^11^	*U*^22^	*U*^33^	*U*^12^	*U*^13^	*U*^23^
S1	0.0359 (2)	0.0934 (4)	0.0619 (3)	−0.0073 (2)	0.00745 (19)	−0.0024 (3)
N1	0.0322 (6)	0.0367 (6)	0.0412 (6)	−0.0031 (4)	0.0117 (5)	0.0009 (5)
C4	0.0378 (7)	0.0337 (7)	0.0434 (8)	−0.0037 (5)	0.0092 (6)	−0.0015 (6)
C3	0.0367 (7)	0.0370 (7)	0.0537 (9)	−0.0041 (6)	0.0171 (6)	−0.0012 (7)
O4	0.0437 (6)	0.0390 (6)	0.0968 (10)	−0.0075 (5)	0.0228 (6)	0.0094 (6)
C6	0.0419 (8)	0.0363 (7)	0.0463 (8)	0.0011 (6)	0.0110 (6)	0.0068 (6)
C7	0.0307 (6)	0.0412 (7)	0.0425 (7)	0.0006 (5)	0.0049 (5)	0.0076 (6)
O2	0.0509 (7)	0.0672 (8)	0.0649 (8)	−0.0050 (6)	0.0300 (6)	−0.0141 (6)
C8	0.0352 (7)	0.0517 (9)	0.0421 (8)	−0.0012 (6)	0.0057 (6)	0.0007 (7)
O3	0.0621 (8)	0.0351 (6)	0.0998 (11)	0.0017 (5)	0.0304 (7)	0.0084 (6)
C13	0.0463 (8)	0.0347 (7)	0.0505 (9)	−0.0031 (6)	0.0150 (7)	0.0036 (7)
C5	0.0394 (7)	0.0338 (7)	0.0413 (7)	−0.0034 (5)	0.0114 (6)	0.0031 (6)
C1	0.0422 (8)	0.0594 (10)	0.0431 (8)	−0.0062 (7)	0.0154 (6)	0.0020 (8)
O1	0.0476 (7)	0.1061 (11)	0.0566 (7)	−0.0012 (7)	0.0266 (6)	0.0016 (8)
C12	0.0402 (8)	0.0503 (9)	0.0498 (9)	−0.0006 (6)	0.0135 (6)	0.0031 (7)
C11	0.0370 (8)	0.0683 (11)	0.0586 (10)	−0.0074 (7)	0.0141 (7)	0.0107 (9)
C9	0.0434 (8)	0.0533 (9)	0.0537 (10)	−0.0066 (7)	−0.0016 (7)	−0.0059 (8)
C10	0.0410 (9)	0.0574 (10)	0.0656 (11)	−0.0153 (7)	0.0025 (8)	0.0059 (9)
C2	0.0475 (10)	0.1065 (16)	0.0468 (9)	−0.0154 (10)	0.0116 (8)	−0.0008 (10)
C14	0.0562 (11)	0.0524 (10)	0.1121 (18)	−0.0215 (9)	0.0171 (11)	0.0127 (11)
C15	0.0886 (16)	0.0700 (13)	0.0914 (16)	−0.0096 (11)	0.0524 (13)	−0.0158 (12)

### Geometric parameters (Å, °)

**Table:**

S1—C3	1.7485 (17)	C13—C5	1.4911 (19)
S1—C2	1.7953 (19)	C1—O1	1.2064 (19)
N1—C1	1.3665 (19)	C1—C2	1.512 (2)
N1—C3	1.3901 (18)	C12—C11	1.389 (2)
N1—C4	1.4631 (18)	C12—C15	1.501 (3)
C4—C5	1.504 (2)	C11—C10	1.371 (3)
C4—H4A	0.9700	C11—H11	0.9300
C4—H4B	0.9700	C9—C10	1.371 (3)
C3—O2	1.1939 (19)	C9—H9	0.9300
O4—C13	1.3342 (18)	C10—H10	0.9300
O4—C14	1.4457 (19)	C2—H2A	0.9700
C6—C5	1.331 (2)	C2—H2B	0.9700
C6—C7	1.474 (2)	C14—H14A	0.9600
C6—H6	0.9300	C14—H14B	0.9600
C7—C8	1.386 (2)	C14—H14C	0.9600
C7—C12	1.403 (2)	C15—H15A	0.9600
C8—C9	1.387 (2)	C15—H15B	0.9600
C8—H8	0.9300	C15—H15C	0.9600
O3—C13	1.1953 (18)		
			
C3—S1—C2	92.82 (8)	N1—C1—C2	112.00 (13)
C1—N1—C3	116.89 (13)	C11—C12—C7	118.10 (16)
C1—N1—C4	122.45 (12)	C11—C12—C15	120.67 (16)
C3—N1—C4	120.67 (12)	C7—C12—C15	121.21 (15)
N1—C4—C5	113.10 (12)	C10—C11—C12	121.87 (16)
N1—C4—H4A	109.0	C10—C11—H11	119.1
C5—C4—H4A	109.0	C12—C11—H11	119.1
N1—C4—H4B	109.0	C10—C9—C8	119.50 (16)
C5—C4—H4B	109.0	C10—C9—H9	120.3
H4A—C4—H4B	107.8	C8—C9—H9	120.3
O2—C3—N1	124.91 (15)	C11—C10—C9	120.04 (15)
O2—C3—S1	124.04 (12)	C11—C10—H10	120.0
N1—C3—S1	111.04 (11)	C9—C10—H10	120.0
C13—O4—C14	116.05 (14)	C1—C2—S1	107.24 (12)
C5—C6—C7	127.12 (13)	C1—C2—H2A	110.3
C5—C6—H6	116.4	S1—C2—H2A	110.3
C7—C6—H6	116.4	C1—C2—H2B	110.3
C8—C7—C12	119.52 (14)	S1—C2—H2B	110.3
C8—C7—C6	120.94 (14)	H2A—C2—H2B	108.5
C12—C7—C6	119.46 (14)	O4—C14—H14A	109.5
C7—C8—C9	120.95 (15)	O4—C14—H14B	109.5
C7—C8—H8	119.5	H14A—C14—H14B	109.5
C9—C8—H8	119.5	O4—C14—H14C	109.5
O3—C13—O4	123.06 (14)	H14A—C14—H14C	109.5
O3—C13—C5	125.24 (14)	H14B—C14—H14C	109.5
O4—C13—C5	111.70 (12)	C12—C15—H15A	109.5
C6—C5—C13	117.15 (13)	C12—C15—H15B	109.5
C6—C5—C4	123.91 (13)	H15A—C15—H15B	109.5
C13—C5—C4	118.92 (12)	C12—C15—H15C	109.5
O1—C1—N1	123.70 (15)	H15A—C15—H15C	109.5
O1—C1—C2	124.30 (15)	H15B—C15—H15C	109.5
			
C1—N1—C4—C5	−60.93 (18)	O4—C13—C5—C4	−7.7 (2)
C3—N1—C4—C5	119.28 (14)	N1—C4—C5—C6	125.99 (15)
C1—N1—C3—O2	−177.60 (15)	N1—C4—C5—C13	−52.85 (18)
C4—N1—C3—O2	2.2 (2)	C3—N1—C1—O1	178.80 (16)
C1—N1—C3—S1	1.36 (16)	C4—N1—C1—O1	−1.0 (2)
C4—N1—C3—S1	−178.84 (10)	C3—N1—C1—C2	−1.4 (2)
C2—S1—C3—O2	178.29 (16)	C4—N1—C1—C2	178.78 (15)
C2—S1—C3—N1	−0.68 (13)	C8—C7—C12—C11	−1.2 (2)
C5—C6—C7—C8	53.3 (2)	C6—C7—C12—C11	−178.06 (14)
C5—C6—C7—C12	−129.92 (17)	C8—C7—C12—C15	−179.69 (17)
C12—C7—C8—C9	0.3 (2)	C6—C7—C12—C15	3.5 (2)
C6—C7—C8—C9	177.09 (14)	C7—C12—C11—C10	1.6 (3)
C14—O4—C13—O3	−5.9 (3)	C15—C12—C11—C10	−179.94 (19)
C14—O4—C13—C5	174.51 (16)	C7—C8—C9—C10	0.3 (2)
C7—C6—C5—C13	−179.58 (15)	C12—C11—C10—C9	−1.0 (3)
C7—C6—C5—C4	1.6 (3)	C8—C9—C10—C11	0.0 (3)
O3—C13—C5—C6	−6.2 (3)	O1—C1—C2—S1	−179.42 (16)
O4—C13—C5—C6	173.39 (14)	N1—C1—C2—S1	0.8 (2)
O3—C13—C5—C4	172.74 (17)	C3—S1—C2—C1	−0.06 (15)

### Hydrogen-bond geometry (Å, °)

**Table:**

Cg1 is the centroid of the C7–C12 benzene ring.

**Table:**

*D*—H···*A*	*D*—H	H···*A*	*D*···*A*	*D*—H···*A*
C8—H8···O2^i^	0.93	2.55	3.379 (2)	148.
C9—H9···Cg1^ii^	0.93	2.90	3.677 (2)	142

Symmetry codes: (i) −*x*+1, *y*, −*z*+1/2; (ii) *x*+1, −*y*, *z*−1/2.
